# State of the Art Review: *Emerging Therapies: The Use of Insulin Sensitizers in the Treatment of Adolescents with Polycystic Ovary Syndrome (PCOS)*

**DOI:** 10.1186/1687-9856-2011-9

**Published:** 2011-08-26

**Authors:** David H Geller, Danièle Pacaud, Catherine M Gordon, Madhusmita Misra

**Affiliations:** 1 Division of Pediatric Endocrinology, Cedars-Sinai Medical Center, David Geffen-UCLA School of Medicine 8700 Beverly Blvd., Rm 4220, Los Angeles, CA 90048, USA; 2 Division of Pediatric Endocrinology, Alberta Children's Hospital, University of Calgary, 2888 Hospital Drive NW, Calgary, Alberta T2N 4Z6, Canada; 3 Divisions of Adolescent Medicine and Endocrinology, Children's Hospital and Harvard Medical School, 300 Longwood Avenue, 333 Longwood-6, Boston, MA 02115, USA; 4 Pediatric Endocrine Unit, Mass General Hospital for Children and Harvard Medical School, 55 Fruit Street, Boston, MA 02114, USA

## Abstract

PCOS, a heterogeneous disorder characterized by cystic ovarian morphology, androgen excess, and/or irregular periods, emerges during or shortly after puberty. Peri- and post-pubertal obesity, insulin resistance and consequent hyperinsulinemia are highly prevalent co-morbidities of PCOS and promote an ongoing state of excess androgen. Given the relationship of insulin to androgen excess, reduction of insulin secretion and/or improvement of its action at target tissues offer the possibility of improving the physical stigmata of androgen excess by correction of the reproductive dysfunction and preventing metabolic derangements from becoming entrenched. While lifestyle changes that concentrate on behavioral, dietary and exercise regimens should be considered as first line therapy for weight reduction and normalization of insulin levels in adolescents with PCOS, several therapeutic options are available and in wide use, including oral contraceptives, metformin, thiazolidenediones and spironolactone. Overwhelmingly, the data on the safety and efficacy of these medications derive from the adult PCOS literature. Despite the paucity of randomized control trials to adequately evaluate these modalities in adolescents, their use, particularly that of metformin, has gained popularity in the pediatric endocrine community. In this article, we present an overview of the use of insulin sensitizing medications in PCOS and review both the adult and (where available) adolescent literature, focusing specifically on the use of metformin in both mono- and combination therapy.

## Background

Recognition of the highly prevalent association between PCOS and insulin resistance (IR) has stimulated research into the mechanism(s) behind this relationship, defining the metabolic, cardiovascular, and reproductive consequences of the IR, and evaluating therapies that target IR. Much of the current therapeutic paradigm incorporating insulin sensitization is derived from studies in adult women; application to the adolescent requires critical evaluation of the data supporting insulin sensitizer use in this age group. Although not intended as a comprehensive review of PCOS therapy, this report will discuss the options available for the treatment of adolescents with PCOS, with focus on the possible efficacy and costs of insulin sensitizing agents in comparison to more traditional therapies for PCOS.

PCOS is a heterogeneous condition affecting 7-10% of women worldwide [1,2], irrespective of ethnic background [3], making it the most common endocrine disorder among reproductive-aged women. The 2003 Androgen Excess Society (AES) consensus required two of the following three criteria as necessary for the diagnosis: hyperandrogenism, ovarian dysfunction (oligo- or anovulation), and/or a polycystic ovary [4]. Summarizing the report of the recent 4^th ^annual meeting of the Androgen Excess and PCOS Society [5], Yildiz and Azziz noted the difficulty in defining certain sub-phenotypes of PCOS, such as women with irregular menstrual cycling and polycystic ovarian morphology without evidence of hyperandrogenism (previously considered essential for the diagnosis).

While hyperandrogenism is central to classically defined PCOS pathophysiology [6-8], and testosterone and DHEA-S are increased in up to 75% of PCOS patients, obesity and IR are frequently associated [9-11]. As many as 60% of women with PCOS have BMI values in the overweight or obese range [2] and 70% demonstrate IR and diabetes beyond that predicted by weight alone [12-14]. Hyperinsulinemia consequent to obesity and IR places women with PCOS at far greater risk to develop type 2 diabetes (T2DM) than healthy controls [15]: 15-36% of all T2DM reported in women, irrespective of age, is found in association with PCOS [14,16-19]. While most PCOS women demonstrate preserved or even exaggerated insulin secretory responsiveness, many PCOS women, particularly those with a family history of T2DM, manifest secretory impairment and glucose intolerance. In addition, the typically gradual transition from impaired glucose tolerance (IGT) to overt T2DM may be accelerated 5 to 10-fold in women with PCOS [20,21]. Legro demonstrated that 40% of women with PCOS had glucose intolerance, and 7.5% of these women manifested frank T2DM, prevalence rates 5-7 fold higher than those reported in population-based studies of women aged 20-44 [22]. 1/3 of women with PCOS fulfill criteria for the diagnosis of the metabolic syndrome (MBS) [11]. These associated metabolic derangements greatly increase a woman's lifetime risk to develop T2DM and cardiovascular co-morbidities [23,24]. Underscoring concerns about the strong association between IR and PCOS, the AES recently recommended that all patients with PCOS be tested for IGT with a 2-h oral glucose tolerance test every 2 years, and annually if evidence of IGT or additional risk factors for emergence of T2DM is identified. Moreover, the AES position statement proposed that PCOS patients with IGT be treated with intensive lifestyle modification and weight loss, and considered for treatment with insulin-sensitizing agents, even before the onset of overt T2DM [25].

### The association between insulin and androgen excesses: history and cellular mechanisms

The association between disordered carbohydrate metabolism and excessive androgen action in women was first reported in 1921 [26] as "the diabetes of bearded women (*diabète des femmes à barbe*)", preceding Stein and Leventhal's formal description of PCOS [27]. A half-century later, researchers described a group of adolescent girls with the constellation of hyperandrogenism, severe IR, and *acanthosis nigricans*, a dermatologic manifestation of hyperinsulinemia (HAIR-AN, or "type A" IR) [28]. A subset of these patients was shown to have mutations of the insulin receptor [29-31]. A second, distinct form of insulin insensitivity associated with androgen excess in post-menopausal women, designated "type B" IR, results from anti-insulin receptor antibodies [32]. Nevertheless, population analyses of PCOS women consistently fails to demonstrate either of these forms of IR as common to the disorder [33].

Burghen and colleagues observed a positive correlation between basal and glucose-stimulated insulin and androgen levels in PCOS, independent of weight, suggesting a causal relationship between hyperinsulinism and hyperandrogenism [34]. An abundant literature corroborates strong associations between insulin and various circulating androgens in PCOS [35-37], particularly free testosterone levels [38-40]. Emphasizing the apparently singular nature of the abnormalities of insulin action in PCOS, a significant inverse relationship exists between insulin sensitivity and testosterone levels for women with PCOS-associated metabolic derangements a relationship independent of BMI [41-43] whose underlying mechanism remains poorly defined.

More recent reports [37,44] confirm a degree of IR in non-obese women with PCOS not found in lean controls. Ehrmann demonstrated that the striking relationship between PCOS androgen excess and MBS can be linked causally to hyperinsulinemia and thus underlying IR [11]; oscillatory glucose infusions verified concurrent defects in β-cell entrainment in women with PCOS [45]. Insulin secretory defects are more pronounced in PCOS women having first-degree relatives with T2DM [21,45], and appear to develop earlier in the evolution of glucose intolerance in women with PCOS than in the general population. Despite these quantitative defects in β-cell function, women with PCOS generally maintain normal glucose tolerance, in contrast to that observed in classical T2DM [46]. That weight reduction alone often fails to resolve the insulin secretory defects in PCOS further emphasizes the uniqueness of the metabolic derangements in this syndrome [36]. The impairment of insulin dynamics found in PCOS thus appears to differ from that observed in T2DM, both with respect to peripheral insulin sensitivity and β-cell secretory capability.

The hyperinsulinemic response to peripheral IR observed in women with PCOS is frequently modest [47,48]. Corroborating data involving more exacting measures of insulin sensitivity are less consistent, with some studies reporting decreased insulin sensitivity in obese, but not lean PCOS, despite comparably elevated insulin levels [47,49]. These discrepancies may reflect differences in methodology used to quantify insulin sensitivity (*e.g., *fasting measures of glucose and insulin [HOMA, QUICKI]), insulin sensitivity index [ISI], minimal modeling of frequently-sampled IV glucose tolerance testing [FSIGT], insulin clamps). Surrogate measures based on fasting insulin and glucose correlate poorly with gold standard techniques such as the euglycemic clamp in the assessment of insulin dynamics in adult PCOS [50], 2-hr glucose levels in adolescents with PCOS [51], and FSIGT in healthy children [52]. Moreover, the excess abdominal fat mass observed in both obese and lean PCOS likely contributes to attenuated insulin sensitivity [53-57].

In response to whole body insulin insensitivity, a more permissive transduction of the insulin signal at the ovarian thecal level may contribute to the pathogenesis of the androgen excess [reviewed in [16]], amplified even in the absence of markedly elevated circulating levels of insulin. Human theca cells possess the full complement of insulin signaling elements and insulin increases thecal testosterone biosynthesis in dose-dependent fashion [58,59]; thus, insulin exerts a direct effect on androgen synthesis. In both obese and lean PCOS, insulin augments *CYP17 *activity, increasing androgen production [60,61]. Serine phosphorylation of both the insulin receptor and regulatory steroidogenic enzymes by as-yet unidentified serine kinase has been proposed to underlie both cellular IR and increased ovarian androgenesis in women with PCOS [62]. Insulin also suppresses hepatic sex hormone binding globulin (SHBG) production [63,64], with a consequent increase in free androgen levels. Centrally, androgen excess may reduce hypothalamic feedback inhibition, resulting in increased GnRH pulsatility, particularly during puberty [65-67]. Increased insulin does not appear to govern gonadotropin secretion directly: PCOS women treated with pioglitazone demonstrate improved insulin sensitivity without alterations in LH pulse frequency or amplitude, or gonadotropin responses to GnRH [68]. Conversely, some data support a blunting of baseline LH and LH pulse amplitude in obese PCOS women, suggesting perhaps that the effect of IR (and consequent insulin excess) is to inhibit LH secretion [69-72].

### The Heritability of PCOS and IR

A genetic etiology for PCOS has been established on the basis of familial clustering [6,21,43-45], with heritability estimates as high as 0.79 [73]. Genetic studies of PCOS typically extend preliminary genome-wide association studies (GWAS), seeking correlations between specific allelic variants of GWAS-nominated genes and PCOS and/or its co-morbidities (*e.g., *IR) [74]. An exhaustive discussion of the candidate genes identified is beyond the scope of this review and few genes have been broadly accepted as causative, due to lack of replication in larger studies [75-80]. The success of these studies has been limited by the lack of consensus in diagnostic criteria resulting in broad phenotypic heterogeneity (described above), the limited sample sizes of many studies, and the difficulty in identifying among kindreds a PCOS phenotype in males and late adolescent women. Concurrent variation at multiple genetic loci (characteristic of the inheritance patterns of many common complex disorders) and lifestyle/environmental factors exerting epigenetic influences on emerging disease further confound efforts to define PCOS heritability.

Family studies of adult female first-degree relatives of PCOS probands demonstrate that the prevalence of PCOS and associated abnormalities of insulin dynamics are increased, consistent with a dominant mode of transmission [81-83]. Kahsar-Miller [84] determined the prevalence of PCOS in first-degree relatives of women with PCOS to be as high as 35% (mothers) and 40% (sisters), 5 times that observed in the general population. Both IR and T2DM are increased in the parents of women with PCOS compared to the parents of women in the general population [85]. Mothers [86] and sisters [87] of PCOS probands exhibit menstrual irregularities, androgen excess, aberrant lipoprotein profiles, and MBS, with profound defects in insulin secretory function and sensitivity [88]. First-degree male relatives of PCOS women also demonstrate increased adrenal androgens (DHEA-S), IR, endothelial dysfunction and MBS [89-91]. Recent cross-sectional studies by Sir-Peterman demonstrated that daughters of women with PCOS manifest increased 2-hr insulin concentrations (despite preserved glucose tolerance) vs. controls across the entirety of pubertal maturation [92,93]. Conversely, Kent observed only late pubertal hyperinsulinemia in girls predisposed to PCOS [94]. Neither of these studies tracked subjects longitudinally into young adulthood; thus, the causal relationship between disordered insulin action and a permanent, post-pubertal state of androgen excess remains unproven.

### Adolescent IR and post-pubertal androgen excess

Although biochemical evidence of ovarian hyperandrogenism in PCOS is detectable shortly after pubertal onset [95], consequent menstrual irregularity is often underappreciated, masked by the frequent acyclicity of post-menarchal adolescence. Thus, oligomenorrhea may escape recognition as pathologic until young adulthood. The physiological IR that typifies mid-pubertal maturation delays timely recognition of metabolic derangements that precede and promote hyperandrogenism [38]. Peri-pubertal metabolic dysfunction is one of the first phenotypic traits observed in adolescent girls who develop PCOS [96,97]. Lewy demonstrated ~ 50% reduction in insulin sensitivity in adolescents with PCOS and concluded that profound metabolic derangements must already exist early in puberty [98]. Apter reported that adolescents with functional ovarian hyperandrogenism (FOH) exhibit disproportionate, early pubertal increases in mean serum insulin levels [99]. Abnormal insulin dynamics are frequently established by the time phenotypic features of PCOS (hirsutism, cystic acne) emerge in late adolescence, irrespective of subject BMI [100]. Studying a cohort of girls with premature adrenarche, whose androgen profiles are reminiscent of PCOS, Ibanez [101-105] observed increased insulin levels and glucose intolerance throughout maturation. Ovarian hyperandrogenism consistently follows menarche, subsequent to the emergence of IR, supporting the premise that IR promotes dysregulated ovarian androgen production [106,107]. In girls predisposed to PCOS hyperinsulinemia precedes androgen excess [92], suggesting the primacy of metabolic dysregulation in the ontogeny of hyperandrogenism. Overweight and obese adolescent girls, possessing higher circulating insulin levels than lean counterparts [54,66,108], also exhibit elevated androgens, further predisposing them to PCOS [109,110].

Earlier recognition of permanent states of hyperandrogenism necessitates consideration of treatment algorithms proposing earlier intervention to prevent establishment of metabolic, cardiovascular, and reproductive *sequelae *that manifest in young adulthood. However, long-term outcomes data to support the most cost-effective management, especially in the adolescent population, are lacking. A variety of therapeutic modalities are currently employed in the treatment of PCOS: oral contraceptives (OCPs), insulin sensitizing medications (metformin, thiazolidenediones), and agents that exert anti-androgen effects (both androgen receptor blockade and 5α-reductase inhibition), alone or in combination (Table 1). Therapy aimed at reducing androgen over-production in PCOS fails to ameliorate co-morbid IR: no significant improvement in insulin dynamics is observed following long-term treatment of adolescents with PCOS with GnRH analogues [111-114]. Similarly, treatment with anti-androgens fails to rectify metabolic derangements in PCOS patients treated [115-117]. Reduction of hyperandrogenemia thus has little effect on IR. Conversely, as will be discussed below, insulin sensitization decreases both androgen and insulin excess, suggesting that, in selected patients with PCOS, insulin excess may be fundamental to the development of hyperandrogenemia.

**Table 1:** Treatment modalities for Polycystic Ovary Syndrome: mechanism of action and desired clinical impact.

Therapy	Mechanism of Action	Regular menses	↓ Androgen levels or effects	Improves insulin sensitivity	Contraception	Metabolic Effects
**Combination E+P pills**	Endometrial changes ↓ GnRH frequency, ↓ LH and FSH ↑ SHBG ↓ androgens (↓ free (active) androgen)	√√	√		√	May be associated with worsened lipid profile, hypertension, decreased glucose tolerance, and prothrombotic effects
**Androgen blockers**	↓ androgen action		√			May be associated with improved lipid profile and blood pressure control
**Weight loss ± insulin sensitizers**	↑ insulin sensitivity ↑ SHBG ↓ androgens (↓ free (active) androgen)	√	√	√		Associated with improved glucose tolerance, lipid profile, and blood pressure control

(Adapted from personal communication from Paul Boepple, M.D.)

Adolescents with emerging PCOS face considerable lifelong morbidity: i) 2/3 of late adolescents with menstrual irregularity due to FOH remain oligomenorrheic into adulthood [118]; (ii) adolescents with PCOS are 4-5 fold more likely to develop MBS than age- and BMI-matched control girls [96]; and (iii) potentially life-threatening cardiovascular dysfunction has its origins during pubertal maturation, and demonstrable by young adulthood [119-121]. The abundant evidence suggesting that IR and resultant hyperinsulinemia facilitate ovarian hyperandrogenism is central to the argument for the use of insulin sensitizers to treat adolescents with PCOS [122], when treatment might preclude adverse metabolic, reproductive and cardiovascular outcomes.

The remainder of this document will: 1) discuss the primary modalities available for the treatment of PCOS; 2) review the literature that compares and contrasts their efficacy, alone or in combination; 3) where available, evaluate the data concerning treatment of a late adolescent PCOS population; and 4) consider whether insulin sensitization, given the role insulin may play in promoting androgen production, should be considered as first-line therapy for the treatment of adolescents with PCOS.

### Modalities for Treatment of PCOS

Therapy for PCOS becomes necessary in adults in order to induce ovulatory cycles and fertility, and to improve cosmetic appearance (*i.e., *reduction of hirsutism and acne). At the same time, it is important to address obesity and associated metabolic complications, which include: 1) endothelial dysfunction and inflammation, 2) an atherogenic serum lipoprotein profile, 3) increased coronary artery calcification, 4) nonalcoholic fatty liver disease and nonalcoholic steatohepatitis, and 5) obstructive sleep apnea. Therapeutic options include lifestyle modification, combine oral contraceptive pills, androgen receptor antagonists, insulin-lowering medications (*e.g., *metformin and thiazolidinediones) (Table 1). In adolescents with PCOS, induction of ovulatory cycles and fertility is of lesser importance, although it is important to induce some element of menstrual cyclicity in order to optimize endometrial health. Improving cosmetic appearance and reduction of weight and obesity- associated metabolic complications are important therapeutic targets.

### Lifestyle modification and weight loss

The first line of therapy in obese adolescents with PCOS is lifestyle modification and weight loss. In addition to an improvement in metabolic co-morbidities of obesity, this would be expected to reduce insulin levels and increase insulin sensitivity, resulting in a decrease in LH and androgen levels. Behavioral, exercise and dietary modifications should therefore be encouraged from the initial encounter, particularly in adolescents, to optimize healthy habits before adulthood, and while these changes are still possible and preventive.

#### Adult women with PCOS

Salmi reported improvement in almost every clinical variable associated with PCOS after weight loss [123], and studies have demonstrated that even modest reductions in weight in the range of 2-7% are successful in improving ovulatory function in at least some adult women with this condition [124-126]. Modest weight reductions with improved fitness are as effective as more drastic reductions in weight to reverse metabolic dysfunction associated with PCOS and ovulatory function in adult women [127]. Recently, Palomba reported a decrease in IR with associated improvement in menstrual cyclicity, fertility, SHBG, and androgen levels in adults with PCOS randomized to either a structured training program or a hypocaloric hyperproteinemic diet [128]. The authors posited that improved insulin sensitivity was integral to these changes albeit brought about by different mechanisms and interventions. In contrast, Hoeger reported increases in ovulation rates only after weight loss [124]. Kiddy similarly reported that weight loss of at least 5% (with a 1000 calorie, low fat diet for 6-7 months) was necessary to increase SHBG, decrease insulin levels and improve reproductive function in adults with PCOS [126]. Whereas the degree of IR in PCOS is greater than that predicted by BMI alone, nearly half of PCOS women are not obese [129] and the degree of visceral adiposity alone is inadequate to explain differences in insulin sensitivity between PCOS and normal women, suggesting that weight loss by itself may be insufficient to improve ovarian function in the sub-population of lean women with PCOS[130].

#### Adolescents with PCOS

Consistent with studies in adults, pediatric studies have demonstrated decreases in adrenal and ovarian androgens with weight loss in both obese pre-pubertal and pubertal girls [131,132], and a 59% reduction in free androgen index with a 122% increase in SHBG levels in obese adolescent girls with PCOS [133]. In the last study, changes were observed despite only modest weight reductions. Importantly, increases in SHBG were much greater in girls who lost weight compared to those who did not.

However, lifestyle modifications are difficult to sustain and are associated with high degrees of recidivism. One study reported a drop out rate of almost 40% in adult subjects enrolled in a study that included intensive lifestyle modification [124]. In a study of adolescents with PCOS, 30% of subjects enrolled into an intensive lifestyle modification program dropped out; 40% attended less than 50% of the sessions and demonstrated no weight change [133]. Therefore, in most instances, pharmacological therapy for PCOS becomes necessary. Available data indicate that in adult women with PCOS, the addition of metformin therapy to lifestyle modification appears more effective than lifestyle changes alone in maintaining weight loss and in ameliorating metabolic complications of obesity [134,135]. In addition, marked improvements in androgen profiles on the basis of a regimented diet and exercise program would be less anticipated in those adolescents with PCOS and normal body composition.

A potential alternative to pharmacological therapy, which we discuss subsequently, particularly in extremely overweight adolescents, is weight reduction surgery. Surgical weight loss procedures have the advantage of more extensive and persistent weight loss than dietary or exercise programs. With the emerging popularity of bariatric surgery to reduce weight in very obese adults, studies have examined the effectiveness of the surgical approach in reducing the severity of clinical features associated with PCOS in these patients. Two studies reported that all adult women with PCOS undergoing gastric bypass surgery resumed menses in the subsequent months, with a significant improvement in hirsutism scores [136,137] and reductions in androgen levels [137]. In one study, about 77% of women reported moderate to complete resolution of hirsutism [136] and in another study, a 48% reduction in hirsutism scores and a 62.5% reduction in free testosterone levels was reported [138]. Bariatric surgery has been successfully implemented in a few hundred adolescents [139]; as more adolescents undergo these procedures, the necessary data regarding their effectiveness in treating associated PCOS will become available. At this time, the possible morbidity associated with this type of surgery limits weight loss surgery to select adolescents with extremely high BMI and associated complications, and centers with an appropriate team-centered approach.

### Use of Oral Contraceptive Pills (OCPs) in PCOS

OCPs are considered to be among the primary treatment options for both adult and adolescent women with PCOS, particularly for those who did not wish to become pregnant [140]. These agents produce regular menstrual cyclicity, lower the risk of endometrial hyperplasia, and dramatically improve acne and hirsutism. Most commonly used OCPs contain both an estrogenic component (typically ethinyl estradiol, in doses ranging from 15 to 50 μg) and a progestin with variable potency and androgenicity [141]. Oral contraceptives improve symptoms through a variety of mechanisms. Estrogens increase the production of SHBG, resulting in a decrease of circulating free androgens, as well as their bioavailability [142]. Progestins protect the endometrium against hyperplasia induced by unopposed estrogen stimulation. The androgenicity of the progestins, mediated through differential androgen receptor binding and blockade [143] or inhibition of 5α-reductase activity [144,145], varies depending on the dosage used and on androgen measurement indices. Some progestins, such as drospirenone and cyproterone acetate, have proven anti-androgenic effects and therefore may yield added benefit in PCOS [146]. Finally combined OCPs suppress luteinizing and follicular stimulating hormones, resulting in reduced ovarian stimulation and androgen production. However, none of these mechanisms directly affect IR [147].

Despite the popularity of OCP use and the number of available combinations, studies contrasting the different agents in head to head comparison are few, their results often equivocal (and contradictory), and few have included an adolescent population. The outcome measures assessed among different studies vary considerably, making the comparison of results difficult. Van der Vange [148] randomized 70 healthy adult women to 7 different OCPs preparations for 6 months; and because the absolute free testosterone levels were similar in all 7 groups, they concluded that these preparations would be of equal benefit in the treatment of hyperandrogenic symptoms. A more recent study compared the effect of 4 OCPs with different progestins on hormonal parameters in 40 women with PCOS over 3 months [149]. They found that all 4 progestins combined with the same dose of ethinylestradiol resulted in significant decrease in testosterone (total and free), androsteredione, dehydroepiandrosterone sulfate; but drospirenone and chlormadinone had more pronounced effects than desogestrel and gestodene. However, clinical effect was not assessed. In a 12-month randomized control trial of adolescents with PCOS testing the efficacy of combined pills with desogestrol or cyproterone acetate as the progestin, the preparations were found to have similar effects on hirsutism and androgen levels [150]. Finally, the benefit of combining GnRH analog with OCPs to suppress LH pulsatility and androgen production has been investigated, particularly with respect to moderate-severe hirsutism (see below); little added benefit was observed despite the marked increase in cost over OCPs alone [39,151].

Independent of their effect in improving the signs and symptoms of PCOS, OCPs possess additional benefits that may support a decision for their use in this age group. The contraceptive potential of birth control pills is a paramount consideration: 1/3 of teenage girls with PCOS reported being sexually active in one recent study [152]. Women with PCOS may be at increased risk of developing gynecologic cancers [153-155], and the use of OCPs reduces the risk of both ovarian [156] and uterine cancer [157] in the general population of women. Conversely, a recent Cochrane review on the treatment options for women with PCOS found insufficient data for the efficacy of OCP in preventing endometrial cancers in this adult population [158].

Regardless of their potential benefits, use of OCPs fails to diminish IR in PCOS and may actually be associated with long-term metabolic derangements, such as glucose intolerance, abnormal lipid profiles and cardiovascular disease. Recent work by Mastorakos showed that a 12-month use of newer OCPs containing either desogestrel or cyproterone as progestin was associated with decreased insulin sensitivity and increase total, LDL and HDL cholesterol and variable changes in triglycerides in adolescents with PCOS [150,159]. Two recent meta-analyses have linked current use of low dose OCPs in women without PCOS to more than a doubling of the risk of myocardial infarction (risk estimates 2.12 (95% confidence interval (CI) = 1.56, 2.86) [160] and 2.48 (95% CI: 1.91-3.22) [161]. Although the use of OCPs is associated with an overall increased risk of adverse cardiovascular events (*e.g., *venous thrombosis and myocardial infarction) among all users, the absolute risk remains minimal in adolescents, even in the sub-population using tobacco [162,163]. While newer OCPs containing less androgenic progestin agents have the potential for less deleterious impact on IR and lipid profile, an insufficient number of adolescent subjects receiving OCPs (and anti-androgen) monotherapy have been studied to draw definitive conclusions about their long-term safety. Moreover, longitudinal studies have not yet been conducted among the population of women with PCOS receiving OCPs, adult or adolescent; thus, the potential exists for the excessive use of OCPs to exacerbate the underlying metabolic derangements prevalent in PCOS, thereby augmenting the subsequent risk for adverse cardiovascular outcomes. Finally, there may be social, ethnic and/or religious stigmata associated with the use of OCPs in some adolescent populations. These concerns have led to a consideration of other approaches for adolescents with PCOS, and specifically, treatment that concurrently targets IR.

### Studies of Insulin Sensitizers and Insulin Lowering Drugs in Adolescents with PCOS

These medications act to reduce insulin levels (metformin) and increase insulin sensitivity (metformin and thiazolidenediones), thus treating the metabolic co-morbidities associated with PCOS and obesity. Reductions in insulin levels effect a concurrent reduction in androgen levels (discussed subsequently) and induce menstrual cyclicity and ovulatory cycles in a majority of those treated. However, recent guidelines recommend that (i) clomiphene (rather than metformin) should be used to induce ovulatory cycles in adult women with PCOS desiring fertility, and (ii) hyperandrogenism causing hirsutism should be treated with estrogen-progestin combination pills, with addition of an androgen receptor blocker after six months if the former are not effective in reducing hirsutism (reviewed in [164]). The role of insulin-lowering agents and sensitizers such as metformin in PCOS may be limited to optimizing weight loss when used with lifestyle modification, and in treatment or amelioration of metabolic complications including hyperlipidemia and reduction of levels of pro-inflammatory cytokines, Additional reasons to consider the use of metformin or thiazolidenediones rather than combined oral contraceptive pills may include the presence of Factor V Leiden mutations, which increase the risk of thromboembolic episodes with estrogen use, or an intolerance, non-adherence, or refusal to use estradiol-progesterone (E_2_-P) combination pills. The paucity of randomized controlled studies of the various therapeutic options in adolescents with PCOS makes it difficult to develop therapeutic guidelines in this younger population. In subsequent sections, we review available data in adolescents, and some data from adults with PCOS, that compare the efficacy of metformin and thiazolidinediones versus combined OCPs in inducing menstrual cyclicity, reducing hyperandrogenism and hirsutism, and effects of these medications on lipid levels and levels of cardiovascular risk markers.

### 1. Metformin

Metformin increases insulin sensitivity in the liver by: 1) reducing gluconeogenic enzyme activities (PEPCK, FBPase, glucose-6-phosphatase), 2) inhibiting hepatic uptake of lactate and alanine, 3) increasing the conversion of pyruvate to alanine, and 4) inhibiting glucose output. The cellular consequence of these AMP-activated protein kinase (AMPK)-mediated effects is alterations in the AMP/ATP ratio. In addition, metformin increases peripheral glucose uptake, decreases fatty acid oxidation and decreases glucose absorption from the gut. Molecularly, metformin-induced phosphorylation modulates the activities of both α1 and α2 catalytic subunits of AMPK, resulting in improved muscle glucose uptake in the presence of insulin. Metformin-induced activation of AMPK may also augment hepatic fatty acid oxidation and improve hepatic insulin sensitivity. In human muscle biopsies, metformin's effect is transduced by phsophorylation of the α2 subunit threonine residue 172, an effect maintained after discontinuation of the medication. However, in mouse skeletal muscle cell lines, metformin-induced phosphorylation of (primarily) α1 Thr172 had no effect on AMP/ATP ratios. Thus, the actual mechanism by which metformin activates AMPK to sensitize target tissues to insulin, or even whether the metformin effect requires AMPK in humans, remains unproven [165].

Knowledge of the effects of insulin on free androgen levels has prompted multiple trials of the efficacy of drugs that increase insulin sensitivity or reduce insulin levels, including metformin, in the treatment of PCOS [166-171]. Most of these studies targeted adult women with this disorder, often yielding promising results with respect to resumption of menstrual cyclicity (in 50-60%), improved percentage of ovulatory cycles, and fertility [reviewed in [170]]. However, a recent large randomized study of more than 600 women reported no improvement in fertility (as assessed by live birth rates) with use of extended release metformin in women with PCOS compared with clomiphene [172,173]. In adults with PCOS, increased abdominal fat, decreased insulin sensitivity, and endothelial dysfunction also contribute to increased cardiovascular risk, and are associated with alterations in circulating levels of adipocytokines; cardiovascular risk markers such as endothelin-1, plasminogen activator-1 (PAI-1), and lipoprotein (a); pro-inflammatory markers such as IL-1, TNF-α, and CRP; and higher neutrophil counts [174-186]. The contribution of decreased insulin sensitivity to increased cardiovascular risk makes a strong case for the use of insulin sensitizers in PCOS in adults for benefits beyond those associated with reproductive integrity. Metformin has been used successfully in adult obese and non-obese women to decrease levels of markers of cardiovascular risk in studies lasting an average of six months [174,176,181,185-188].

Studies in adolescents with PCOS have been limited, and data on the benefits of therapeutic intervention on restoration of menstrual cyclicity less convincing than in adults [174,189-191]. Longitudinal data are sparse regarding the effects of decreased insulin sensitivity and increased abdominal fat on long-term cardiovascular outcomes in teenagers with PCOS, and few studies have examined the relationship between cardiovascular risk factors and the use of metformin in this younger population. Recent work by Ibanez indicates that relatively lean girls with hyperinsulinemic hyperandrogenism have a dyslipidemic profile, with high levels of triglycerides and other atherogenic lipids [192]. Similarly, an increased prevalence of the metabolic syndrome in adolescent girls with PCOS compared with weight matched controls reported by Coviello included evidence of abnormal lipid profiles [96].

A recent survey indicated that 30% of pediatric endocrinologists consider metformin appropriate treatment for adolescents with PCOS, and 68% for obese adolescents with PCOS [193]. The following sections briefly review some adult and available adolescent data regarding efficacy of insulin sensitizers in treating those with PCOS. The bulk of available data in adolescents derives from studies by Ibanez in relatively lean girls with a history of precocious pubarche, hyperinsulinemic hyperandrogenism, and post-pubertal features consistent with PCOS, studies which remain to be replicated [55,189,192,194-197].

#### Androgen-lowering effects of metformin

In both lean and obese women with PCOS, metformin (i) decreases insulin levels in association with decreases in clinical indices of ovarian cytochrome P450c17 activity, and (ii) increases SHBG levels, resulting in decreases in free testosterone [60,61,63,64]. Long-term metformin therapy may also decrease the activity of several other adrenal steroidogenic pathway elements [198,199]. In addition, metformin use is associated with increases levels of IGFBP-1 (which may be a consequence of a reduction in insulin levels), with the resultant decrease in IGF-1 bioavailability possibly decreasing the stimulatory effects of IGF-1 on ovarian steroidogenesis. However, Munir demonstrated that modulation of human theca P450c17 *in vitro *is transduced exclusively through insulin receptors, rather than IGF-1 receptors [59], making less clear the role of IGF-1 in governing thecal androgen production at the whole body level.

Studies indicate that the degree to which metformin therapy is effective in improving the androgenic and metabolic profile in adolescents with PCOS may be related to (i) dose used, particularly when metformin is used as monotherapy, necessitating higher doses [191,200-202]; (ii) the agent with which metformin is combined, estrogen-progestin combination pills or anti-androgenic agents such as flutamide, spironolactone, and cyproterone acetate [115,116,203-210]; and (iii) the characteristics of the PCOS patient being treated, whether obese or lean, hyperinsulinemic, or normoinsulinemic [191,194,200,202,211,212]. We will consider each of these aspects individually:

#### Metformin monotherapy

A dose of 1.5-2 g per day appears necessary for clinical effectiveness when metformin is used alone [201], the efficacy dependent on the outcome measure chosen. Arslanian reported decreased insulin sensitivity in fifteen obese adolescents with PCOS in whom metformin therapy (850 mg, twice daily) was associated with an improvement in glucose tolerance and a decrease in testosterone levels [191]. Six of the study subjects had simultaneous improvements in menstrual cyclicity. Of note, these girls also had significant decreases in BMI (mean decrease of 1.4 kg/m^2^) over the three-month study period. In another study of eleven obese girls with PCOS, metformin use was associated with a downward trend in circulating testosterone concentrations and a decrease in total cholesterol levels, even after adjusting for weight loss [213]. Although a daily metformin dose of 1.5 g (500 mg three times daily) was effective in two of the eleven girls, nine required an increase to 2.55 g (850 mg three times a day) after 8-10 weeks on the lower dose. Ninety percent of those treated resumed regular menses, and in 39% of the follow up visits in girls with regular menses, the cycles were ovulatory, based on luteal phase (day 21) progesterone levels. The proportion of girls resuming menses in this study was higher than that observed in other studies of women with PCOS receiving metformin, in which menstrual cyclicity was restored in fewer than 50% overall [202,204,214,215]. A third uncontrolled study in 18 adolescent girls 15-18 years old similarly reported a reduction in levels of androgens and resumption of menstrual cyclicity and ovulatory cycles in all 16 girls who tolerated 1700 mg of metformin daily. Girls in this study also had a reduction in BMI that was maintained (along with regular and ovulatory menstrual cycles) six months after the end of metformin treatment [216]. The absence of a control group and significant weight loss in subjects in these studies make these data difficult to interpret in terms of metformin's efficacy independent of weight loss.

Similar results from a placebo-controlled study by Bridger appear to confirm beneficial effects of metformin monotherapy, at least in the short term [200]. In this study, 22 obese adolescents with hyperinsulinemia and PCOS were randomized to receive metformin (1.5 g per day) or placebo for a twelve-week period. The group receiving metformin had significant decreases in levels of testosterone and increases in HDL-cholesterol, in the absence of changes in insulin sensitivity or BMI. Because insulin sensitivity did not significantly change with metformin, a direct effect of metformin on androgen secretion could not be ruled out. However, in an uncontrolled study, Glueck reported that metformin therapy associated with caloric restriction for 12 months in 35 adolescents with PCOS was associated with a 4.4% decrease in weight, as well as decreases in insulin, HOMA-IR (an index of insulin sensitivity derived from fasting glucose and insulin values), cholesterol and TG, and improved menstrual function, with regular monthly cycles increasing from 22% to 74% [217].

Lower doses of metformin have proven effective in non-obese adolescents with PCOS. Ibanez and colleagues reported decreased ovarian hyperandrogenism, hyperinsulinemia, and hirsutism, as well as improved menstrual cyclicity and lipid profiles in ten non-obese girls with hyperinsulinemic hyperandrogenism treated with as little as 1.25 g of metformin daily [197]. Leukocytosis, another pro-inflammatory surrogate marker observed in adolescents with hyperinsulinemic hyperandrogenism, also normalizes with metformin monotherapy (vs. no medication) in a randomized study design [174].

Metformin use is associated with gastrointestinal side effects, which can be minimized by slow titration of the medication to the desired dose over a one-month period. In none of the studies presented above were severe adverse effects with metformin observed; specifically, lactic acidosis has not been reported in this population. Compliance with the higher doses required in some teenagers may be an obstacle to efficacy of metformin therapy, given the potential for initial gastrointestinal side effects [200]. Extended-release metformin may be useful in such instances.

#### Metformin (with or without anti-androgens) versus estrogen-progestin combination pills)

E_2_-P combination pills are the treatment of first choice in adolescents with PCOS (see above), particularly when efforts at weight loss, including reduction in caloric intake and increased physical activity, fail. Although exogenous estrogen decreases free androgen levels by increasing levels of SHBG and suppressing ovarian secretion of gonadal steroids (both estrogen and androgen), it neither increases insulin sensitivity nor decreases inflammatory mediators, adipose tissue or adipose-derived adipocytokines, based on studies in adults [55,150,189,210,218,219], and may cause an increase in lipids. In adolescent girls with PCOS, Mastorakos demonstrated an increase in levels of total, LDL- and HDL-associated cholesterol with combined E_2_-P containing pills, and a tendency towards increasing triglyceride levels with use of cyproterone acetate-containing combination pills [150]. The same group later reported increased IR after 12 months of both E_2_-P- and cyproterone acetate-containing combination pills. The later formulation also resulted in hyperinsulinemia [159].

An important question is whether metformin can improve menstrual cyclicity and hirsutism scores in PCOS without predisposing to the deleterious metabolic effects attributed to use of combined oral contraceptive agents. To answer this question, Hoeger randomized 43 adolescent girls with PCOS to either lifestyle intervention, estrogen-progestin (ethinyl estradiol + desogestrel) combination pills, metformin (1700 mg daily), or placebo for a six-month period, and demonstrated that groups randomized to lifestyle intervention and the combination pills (but not metformin) had increases in SHBG levels and reductions in free androgen index and in PAI-1 [133] despite only non-significant changes in weight in all groups. Hirsutism scores did not change in any group. Among girls not receiving OCPs, the frequency of menses did not differ between groups; observed menses were ovulatory 75% of the time in the metformin group, as opposed to 60% of the time in the group receiving combined pills and 50% of the time in the group randomized to placebo. However, use of OCPs was associated with a 14% increase in total cholesterol and 40% increase in high-sensitivity CRP, whereas, these adverse metabolic effects were not observed in the other groups, with the metformin group demonstrating a 25% decrease in triglyceride levels. Unlike the previous study, use of combined pills did not result in hyperinsulinemia. Metformin use was associated with a significant decrease in glucose levels, but insulin levels did not change. These data suggest that in obese adolescents with PCOS, OCPs and lifestyle intervention have beneficial effects on androgen levels. However, combination pills affect cardiovascular risk markers adversely, while metformin has beneficial effects on both lipids and glucose levels.

In contrast to these data, Allen found concurrent decreases in both IR (measured by fasted baseline glucose/insulin ratios and QUICKI derivation) and testosterone levels in 35 obese PCOS girls randomized to either metformin or an OCP for six months [220]. Significantly, both cohorts lost weight (mean BMI decrease of 1.5 kg/m^2 ^in the estrogen/progestin group and 1 kg/m^2 ^in the metformin group). The authors concluded that the two drugs produced similar benefits for the cardinal features of PCOS in adolescents.

Other data comparing combine OCPs and metformin derive from Ibanez' studies of non-obese hyperinsulinemic, hyperandrogenic adolescents. In 32 such adolescents with a mean age of 15 years and a BMI of 22 kg/m^2^, Ibanez and de Zegher [189] found low baseline adiponectin levels and increased abdominal fat. Over 3-9 months in a randomized study design, E_2_-P (ethinyl estradiol + drospirenone) combination pills further decreased adiponectin levels while increasing both triglyceride levels and central adiposity. Conversely, metformin (850 mg/day) in combination with low-dose flutamide (62.5 mg/day) reversed these values towards normal, with a 4-kg mean decrease in fat mass and a commensurate increase in lean mass. These authors also observed that metformin attenuated the pro-inflammatory state in young women with hyperinsulinemic hyperandrogenism, resulting in reductions in IL-6 and CRP levels and neutrophil counts, whereas use of E_2_-P combination pills aggravated the pro-inflammatory state [174,189,221,222]. Whereas metformin alone did not significantly reduce the free androgen index in obese adolescents, the combination of metformin and flutamide was as effective as E_2_-P combination pills in reducing the hyperandrogenism associated with PCOS [133]. In addition, metformin was superior to E_2_-P in increasing insulin sensitivity, while decreasing dyslipidemia, anovulation, body adiposity, and the pro-inflammatory state associated with PCOS. It should be noted that the population of girls studied by Ibanez and colleagues all had histories of precocious adrenarche, perhaps suggestive of a unique sub-class of PCOS patients.

Further studies are necessary to determine whether or not metformin exerts beneficial effects beyond those of combined OCPs alone, in the population of adolescent girls with PCOS. Although definitive data from well-controlled trials are lacking, reports suggest the cosmetic result achieved with OCPs may be superior to that of metformin in the treatment of hirsutism in adolescents with PCOS, at least over a prolonged period. It remains to be definitively determined whether the superior metabolic effects achieved with metformin (in contrast with combination pills) in adolescents with PCOS can be sustained and over what duration these effects persist. The re-emergence of phenotypic and metabolic abnormalities following discontinuation of medication is an important consideration, as recent data suggest a loss of the benefits of metformin almost immediately after cessation of therapy [195,197,223]. This may be of particular importance in adolescents in whom compliance with medical therapy is often problematic.

#### Metformin in combination with estrogen-progestin combination pills or anti-androgens

In adult women with PCOS, adding metformin to a combination OCP decreases IR, as well as androgen levels. However, the anticipated correction of deranged lipid profiles and abdominal obesity through metformin use appears to be blunted [209,224,225]. In 36 adolescent obese girls with PCOS randomized for six months to a combined pill (ethinyl estradiol + drosperinone) with lifestyle intervention and metformin (2000 mg daily) or to an OCP with lifestyle intervention and placebo, weight reductions, increases in SHBG, and decreases in free androgen index, and decreases in hirsutism scores occurred in both groups, but did not differ between groups [133]. Total cholesterol increased in both groups, but the metformin group had greater increases in HDL. No changes in insulin or glucose occurred in either group. Data from this study suggest only modest metabolic benefits of adding metformin to a regime of a combined pill with lifestyle intervention in obese adolescents with PCOS.

In contrast, in 31 non-obese girls with hyperinsulinemic hyperandrogenism (average age, 16 years), random assignment to metformin-treated or not to a protocol consisting of flutamide and ethinyl estradiol + drospirenone over a 3-month period resulted in a decrease in IL-6 and an increase in adiponectin levels in the group receiving metformin [222]. The authors examined the effects of randomly withdrawing metformin use or not in 42 lean young women (average age, 19 years), and again observed beneficial effects of metformin. Similarly, addition of metformin-flutamide led to normalization of leukocyte and neutrophil counts in 41 young women (average age, 18 years), in whom prior use of ethinyl estradiol-drospirenone had effected an increase in these inflammatory markers [174]. These women had improvements in insulin sensitivity, dyslipidemia, ovulatory patterns, and adiposity indices as a consequence of the addition of metformin-flutamide to a regimen of OCP monotherapy. These data suggest greater benefit when metformin is added to a regime that includes combination pills in non-obese adolescents and young women with PCOS.

The type of progesterone in the E_2_-P combination pill used in conjunction with insulin sensitizers may also exert variable effects on body composition. Ibanez and de Zegher reported on 29 non-obese young women with PCOS (~ 20 years of age) receiving metformin+flutamide combination therapy. When switched from a combination pill with gestodene to one containing drospirenone, these patients exhibited decreased abdominal fat mass and increased lean mass without changes in overall body weight [221]. Of note, both gestodene and drospirenone are newer synthetic progestins. As a class, progestins bind weakly to the androgen receptor, resulting in variable androgenic effects. Whereas gestodene has minimal androgenic properties, drospirenone exerts no androgenic effects, and in fact antagonizes the androgen receptor after binding (although in doses higher than those used in combination pills). Drospirenone also has anti-mineralocorticoid effects and is therefore thought to minimize the water retention and breast tenderness associated with combination pills.

Anti-androgens such as spironolactone, cyproterone acetate, and flutamide are commonly administered to address the androgen excess features of PCOS. The anti-androgenic effects of flutamide, a non-steroidal androgen receptor antagonist, appear superior to those of spironolactone or cyproterone acetate (the latter not available in the U.S. at present) [203,226]. The combination of metformin with anti-androgens appears promising based on a handful of recent studies in lean [206] and obese [205] young adult women with PCOS, as well as in lean adolescent girls with hyperinsulinemic hyperandrogenism [174,189,190]. This combination effectively decreases abdominal obesity and levels of inflammatory markers in these limited studies. When used alone and in high doses, flutamide can be hepatotoxic; however, flutamide has a good safety profile when used in polytherapy and in lower dosages [116,227]. A combination of low-dose flutamide (62.5 mg) and metformin (850 mg) appears both safe and effective in adolescents and young women, in durations ranging from 3-54 months (mean, 19 months), with preservation of normal liver function [228]. Thus, data from the Ibanez group suggest that low-dose polytherapy provides both an enhanced safety profile and an expanded spectrum of clinical benefit, when contrasted with high-dose monotherapy, owing to the metabolic corrections exerted by the metformin [190]. Lastly, some have endorsed the use of finasteride, a 5-α reductase inhibitor that blocks conversion of testosterone to its dihydrotestosterone metabolite in skin target tissue, to ameliorate the dermatologic effects of androgen excess in adults. This medication remains off-label for adolescents, alone or in combination with metformin or other insulin sensitizers. It is mandatory to exclude pregnancy in those adolescents whose treatment regimen includes the use of anti-androgens, due to their potential for precluding proper virilization of male fetuses. Even more important is the need to emphasize ongoing use of contraception, given the increase in ovulatory rates as a result of metformin use.

#### Metformin use in obese versus lean, and hyperinsulinemic versus normoinsulinemic PCOS

Silfen [100] observed that lean adolescents with PCOS had lower insulin levels, increased insulin sensitivity, and a more favorable lipid profile than did obese adolescents with PCOS. Conversely, levels of adrenal androgens were higher in the lean PCOS subjects. Given these differences, one might anticipate differences in individual responses to metformin as a function of body composition. However, all studies to date report beneficial effects of metformin in lean as well as obese adolescents with PCOS [55,191,195,213].

Regrettably, there is a paucity of data regarding the use of metformin in hyperinsulinemic versus normoinsulinemic adolescents with PCOS, and it remains unclear whether metformin use should be restricted to adolescents with PCOS (both obese and lean) with biochemical or phenotypic (*i.e., *acanthosis nigricans) evidence of hyperinsulinemia. Ibanez and colleagues define hyperinsulinemia as a peak insulin of > 150 μU/mL during a 2-hr oral glucose tolerance test and/or mean serum insulin levels of > 84 μIU/mL during standard oral glucose tolerance testing [54,55,104,189,190,192,195,206,221-223,229-232]. Vuguin [233] and Silfen [234] demonstrated good correlation between fasting glucose:insulin ratios of < 7 mg/10^-4 ^IU and IVGTT measures of IR in adolescents with premature adrenarche, substantially greater than the figure determined for IR associated with adult PCOS (approximately, 4.5 mg/10^-4 ^IU) [235].

Significant effects of metformin on hirsutism scores and ovulation were found in lean, hyperinsulinemic women with PCOS, and a decrease in DHEA-S levels in lean PCOS, regardless of insulin levels [211]. In overweight and obese women with PCOS, significant effects were observed on waist-hip ratio in the normoinsulinemic overweight women, and on menstrual cyclicity in normoinsulinemic obese women. Although these data suggest beneficial effects of metformin on body composition and menstrual function in normoinsulinemic overweight and obese adults, it is not known whether these data can be extrapolated to adolescents, and in particular to lean normoinsulinemic adolescents. Further studies will be necessary to address these specific questions in adolescents with PCOS. In conclusion, use of low-dose polytherapy may be superior to high-dose monotherapy with respect to efficacy and safety profile; however, further studies in adolescents with PCOS are necessary before definitive recommendations can be developed.

#### Prevention of PCOS with metformin (earlier intervention)

Girls at higher risk to develop PCOS, such as those with low birth weight and precocious pubarche [196,236], have a slowing of progression of endocrine-metabolic perturbations associated with PCOS when metformin is started during either the pre-pubertal or early post-menarchal period [223,237]. This observation has been exploited to propose a benefit to early intervention in high-risk groups predisposed to PCOS. Of greater concern are data suggesting that these benefits are lost almost immediately following discontinuation of metformin [195,197,223].

### 2. Thiazolidenediones

Thiazolidenediones act as insulin sensitizers through their activation of the nuclear receptor PPAR-γ, leading to increased production of insulin-sensitive adipocytes and increased glucose uptake in these cells, increased secretion of adiponectin, and decreased secretion of pro-inflammatory cytokines. Recent data in adult women with PCOS suggest that thiazolidinediones exert additional benefit with respect to hyperandrogenism, IR, anovulation, and inflammatory mediator levels, in both lean and obese women with PCOS [212,238-242]. These benefits are observed despite increases in weight, BMI, and waist to hip ratio in those treated [239]. Seto-Young and colleagues have proposed that the effects of thiazolideindiones may be independent of the effects on insulin secretion [243]. These authors reported that PPARγ-agonists stimulate IGFBP-1 and progesterone production, directly decrease estrogen and testosterone secretion, and concurrently antagonize the insulin-induced enhancement of estrogen and testosterone secretion in cultured human ovarian cells. The thiazolidenedione, pioglitazone, has been shown to ameliorate the signs and symptoms of PCOS in a cohort of women who failed a previous trial of metformin [244]. These medications have not been rigorously studied in adolescents, in either traditional states of IR or in late adolescent girls with unambiguous PCOS; thus, they remain "off label" for use in this age group. Despite proven efficacy in mitigating both IR and androgen excess while restoring menstrual cyclicity in adult women with PCOS [245,246], FDA concerns led to the removal of the vanguard thiazolidenedione, troglitazone, from the market. Concerns regarding potential adverse cardiovascular events in T2DM patients taking thaizolidenediones (*e.g., *Avandia, [rosiglitazone]) resulted in the recent addition of a black box warning to the package inserts for that agent, thus reinforcing the need for caution when considering use of this class of medication, in both adolescents and women with PCOS. Concerns about hepatotoxicity and peripheral lipogenetic effects must be addressed before the use of these medications in adolescent girls can be endorsed without reservation [241,243]. Of note, one case report has indicated efficacy of pioglitazone, but not metformin, in ameliorating insulin resistance, hyperinsulinemia, and hyperandrogenism, and in resumption of menses in two sisters with Dunnigan-type familial partial lipodystrophy [247].

## Conclusion

PCOS is a multi-phenotypic disorder characterized by androgen excess and menstrual acyclicity, with demonstrable biochemical aberrations during pubertal maturation, and clinical manifestations shortly after pubertal completion. A variety of therapies may rectify both the biochemical derangements and clinical features (Table 1) of PCOS, and many patients are treated with polytherapy to address the multiple facets of the syndrome. Women with PCOS have abnormalities of insulin secretion and action, and underlying IR has been proposed to be fundamental to the development of ovarian hyperandrogenism. Application of therapies to diminish IR and consequent hyperinsulinemia has gained increasing support as first-line therapy. As listed in Table 2, arguments can be made in support of and against the use of insulin sensitizers in the treatment of adolescent PCOS. Studies demonstrating long-term efficacy and safety of metformin and the thiazolidenediones in the adolescent age group are lacking, having been chiefly limited to adult women with PCOS, particularly those with impaired glucose tolerance and overt T2DM [248]: these are clearly essential in order to promote their use as therapeutic options for adolescents with PCOS. The paucity of data on the effects of these modalities in the treatment of adolescent PCOS highlights the need for well-designed, controlled studies to optimize treatment algorithms for this disorder in the late teen and young adult. The variability of phenotypic expression within the syndrome further confounds advocacy of a single treatment regimen, the determination of which requires individualization to address the specific presenting complaint(s) of the adolescent patient. Additionally, future individualization of treatment options will rely increasingly on pharmacogenomic models that evaluate putative genetic determinants of metformin response (as described in adult type 2 DM patients) [249-252], to improve prediction of therapeutic efficacy. To date, large-scale studies targeting the prevalence of specific metformin response elements have not been undertaken in either adolescent or adult PCOS populations. Finally, when treating teenagers, a comprehensive approach is imperative, one that considers important ancillary issues such as prevention of pregnancy in those who are sexually active, as well as the need to stress lifestyle counseling for those with significant obesity and/or IR. Although beyond the scope of this review, the use of cosmetic measures to combat hirsutism and dermatological approaches to address acne are common practice and serve as useful adjuncts to the specific medical therapies for PCOS. Other cosmetic concerns, particularly regarding the increased BMI, are of paramount importance in this adolescent patient cohort, with serious negative effect on an affected teenager's quality of life [253,254]. The insufficiency of validated data concerning the use of the insulin sensitizers in adolescent PCOS justifies the continued designation of these medications as "off label" in this age group, which thus cannot be recommended as first-line therapy at the present time.

**Table 2:** Pros and cons of insulin sensitizer therapy in PCOS

PROS	CONS
Reduces insulin resistance and addresses an important component of the pathophysiology of PCOS Useful for treating hyperglycemia in patients with PCOS-associated type 2 DM	Insulin-sensitizing effect may not persist after discontinuing medication
Metformin may cause weight reduction and is associated with improvement in lipid profile	Weight reduction is minor with metformin; TZDs may cause weight gain and peripheral lipogenesis
	
	Cosmetic improvements with insulin sensitizers may be less marked than with E_2_-P combination pills
	Insulin sensitizers may induce ovulation with risk of unwanted pregnancy unless used with contraception
An option in patients with Factor V Leiden mutations and other risk factors for coagulopathy in whom E_2_-P combination pills may be contraindicated	
Potential for use in adolescents with lean PCOS in whom lifestyle modification is likely to be ineffective	Lean PCOS responds well to conventional E_2_-P combination pills in conjunction with anti-androgen medications
Excellent safety profile for metformin, with few side effects reported	Insulin sensitizers are potential teratogens Select patients may require frequent monitoring of liver and renal function TZDs have been associated with adverse cardiovascular events in adult patients
	Insufficient studies of efficacy and long term safety of insulin sensitizers in adolescents

## Summary

### Background

1. PCOS, a heterogeneous disorder characterized by cystic ovarian morphology, androgen excess, and/or irregular periods, emerges during or shortly after puberty.

2. Insulin resistance and consequent hyperinsulinemia are highly prevalent and facilitate the formation of excess androgen.

### Lifestyle Changes

1. Lifestyle changes involving behavioral, dietary, and exercise regimens should be considered as first line therapy for weight reduction and improvement of insulin levels in obese adolescents with PCOS.

### OCPs

1. OCPs, the traditional treatment option for both adult and adolescent women with PCOS not wishing to become pregnant, restore menstrual cyclicity and reduce signs of androgen excess without improving IR.

2. Studies on the risk-benefit profiles for different OCPs provide equivocal results with regard to hirsutism, acne, and menstrual dysfunction, and lack sufficient data on their efficacy and safety in the adolescent population.

3. Cardiovascular risk markers, often high in the PCOS population, may be increased by OCPs.

### Metformin

1. Several, although not all, studies of adult women with PCOS treated with metformin demonstrate promising results for resumption of menstrual cyclicity and ovulation, restoration of fertility, improved insulin dynamics, adipocytokine and inflammatory mediator profiles, and cardiovascular indices.

2. The benefits to adults with PCOS in improving hirsutism scores, menstrual cyclicity, and metabolic status depends on whether metformin is used as monotherapy or in combination with anti-androgen and/or OCP, as well as the insulin sensitivity status of the patient.

3. In adolescents with PCOS, little is known about the safety and efficacy of metformin, either in mono- or combination therapy.

### Other

1.	Thiazolidinediones may provide reproductive, metabolic, and cardiovascular function benefits to adult women with PCOS in whom previous metformin therapy failed.

2.	Thiazolidinediones remain off-label in adolescents, due to lack of evidence on efficacy and safety.

## Competing interests

The authors have no competing financial or other interests to declare in relation to this manuscript.

## Authors' contributions

DHG participated in the planning, implementation, writing, review and editing of the manuscript. DP participated in the planning, implementation, writing, review and editing of the manuscript. CMG participated in the review and editing of the manuscript. MM participated in the conception, planning, implementation, writing, review and editing of the manuscript. All authors have read and approved the final manuscript.
